# Linkage analysis of a derived glucose phenotype in the Genetic Analysis Workshop 13 simulated data using a variety of Haseman-Elston based regression methods

**DOI:** 10.1186/1471-2156-4-S1-S6

**Published:** 2003-12-31

**Authors:** Heather J Cordell, Joanna MM Howson, David G Clayton

**Affiliations:** 1 University of Cambridge, Department of Medical Genetics, JDRF/WT Diabetes and Inflammation Laboratory, Cambridge Institute for Medical Research, Wellcome Trust/MRC Building, Addenbrookes Hospital, Hills Road, Cambridge CB2 2XY, United Kingdom

## Abstract

A variety of Haseman-Elston type regression procedures were used to perform a genome scan across five chromosomes, using replicates 1–5 of the Genetic Analysis Workshop 13 simulated data. The traits of interest were variables corresponding to 'baseline' and 'slope' effects derived from the fasting glucose phenotypes. Performance in terms of detecting the locations of known trait loci was poor for all methods, even when all five replicates were combined to produce a large data set (9230 sib pairs). All methods performed well, however, when applied to new simulated data in which the true genetic effects were allowed to explain a greater proportion of the overall variance.

## Methods

Haseman-Elston sib-pair regression procedures were used to perform genetic linkage analysis on chromosomes 1, 2, 3, 9, and 21 in replicates 1–5 of the Genetic Analysis Workshop (GAW13) simulated data. All of these chromosomes, except chromosome 2, were known from the 'answers' to contain trait loci that contributed in some way to fasting glucose. This subset of chromosomes analyzed did not contain all loci involved in fasting glucose levels, but did include 8 out of the 12 loci influencing baseline effects (including the two loci with the largest baseline effects, b14 and b15) and two out of the four loci influencing slope effects (s4 and s6). Independent families were selected for analysis by choosing the largest nuclear family from each pedigree. Multipoint identity-by-descent (IBD) sharing probabilities for each sib pair within a family were calculated from the complete genotype data using the program GENEHUNTER. Trait values were calculated from the complete phenotype data for each sib as described below. Four different regression procedures were used to examine the relationship between the trait values and IBD sharing: the original Haseman-Elston method [1] in which the sib-pair trait difference squared is regressed on the mean IBD sharing, the Haseman and Elston revisited method [2] in which the mean-corrected sib-pair trait cross-product is regressed on the mean IBD sharing, the unified Haseman-Elston method [3] in which the test statistic is derived using appropriately weighted contributions from the sib-pair trait difference squared and the mean-corrected sib-pair trait sum squared regressions respectively, and an IBD regression method [4] in which IBD measures are regressed against trait values as opposed to the other way around. This has the advantage of being particularly appropriate when applied to sib pairs that have been selected according to their trait values [5]. For this last method, the IBD sharing parameter *p *is modelled as a linear function of the sib-pair trait difference squared, sibs are assumed to share 0, 1, or 2 alleles IBD with probability (1 - *p*)^2^, 2*p*(1 - *p*), *p*^2 ^and an appropriate likelihood is calculated [4]. All methods made use of all possible sib pairs in a sibship and corrected for the resulting non-independence of pairs within a sibship by use of Wald tests with robust information-sandwich estimators of the variances [6,7] as opposed to likelihood ratio tests. These robust information-sandwich tests are available as a standard option when carrying out regression analysis in the statistical software package STATA [8].

The trait measures α and γ used in the regression procedures were derived by fitting the model

*y*_*it *_= α_*i*_+ β^T ^*x*_*it *_+ γ_*i *_(*a*_*it *_- 20)     (1)

using the statistical software package STATA [8], where *y*_*it *_is the fasting glucose value for person *i *at examination time *t*, *x*_*it *_is a vector of covariates for person *i *at examination time *t *(here chosen as log(weight), log(height), gender, and the interactions gender*log(weight) and gender*log(height)), and *a*_*it *_is the age of person *i *at examination time *t*. This model assumes that fasting glucose is determined by a baseline value α_*i *_specific to each person at age 20, effects due to covariates (corresponding to the vector of coefficients β) that do not vary with time or age, and a person-specific slope effect γ_*i *_that allows fasting glucose to increase or decrease linearly with age. Note that this model does not correspond to the model that was actually used to generate the data, which in fact takes the more complicated form



Although this more complicated model (2) could, in principle, be fitted, in real life one would be unlikely to propose such a model without prior knowledge of the 'answers' or some other belief concerning the underlying biological process. We therefore choose to fit model (1) as opposed to (2), since this reflects the model that would be more likely to be assumed and hence the procedure that would be more likely to be followed in practice.

## Results and Discussion

**Figure 1:**
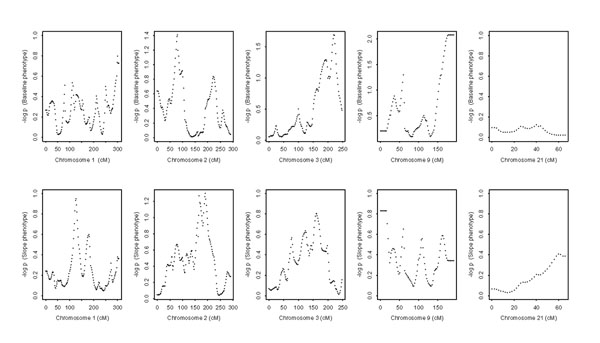
Results (-log *p*-value) for unified Haseman-Elston analysis of derived baseline and slope phenotypes.

Initially the analyses were performed for each replicate separately, but this did not succeed in localizing any of the known genetic effects to their correct locations. The five replicates were therefore pooled and analyzed together to see if the larger ensuing sample size (9230 sib pairs) would help in the detection of the trait loci. Figure 1 shows the results for the baseline (α) and slope (γ measures using the unified Haseman-Elston method [3]: similar results were produced by the other methods (data not shown). Even with such a large sample size, there is little evidence for or localization of any of the known trait loci: the results on chromosomes 1, 3, 9, and 21 are essentially indistinguishable from those on chromosome 2 on which it is known that no trait loci reside.

**Figure 2:**
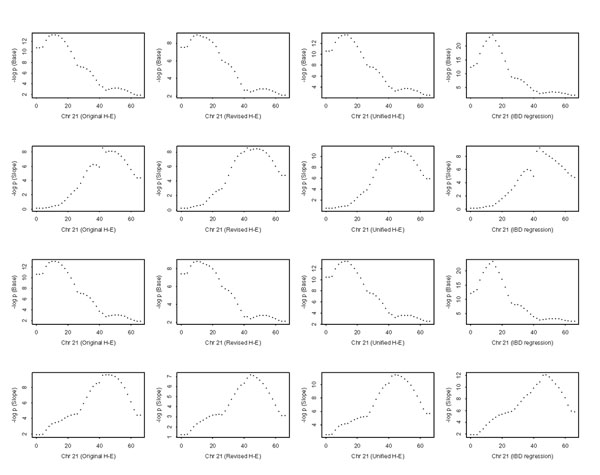
**Results (-log *p*-value) for new simulated data. **Rows 1 and 2 correspond to data simulated under model (1) and rows 3 and 4 to data simulated under model (2). Derived baseline and slope phenotypes were analyzed using four different regression methods.

These disappointing results could be due to the relatively small proportion of the overall variance that is accounted for by each trait locus, and/or to the fact that our phenotype measures α and γ are very poor measures of the true baseline and slope effects due to the difference between the generating model (equation (2)) and that assumed (equation (1)) when deriving α and γ. To assess the performance of our procedure in a situation where the true generating model does in fact take the form of equation (1), we used the GAW13 simulated data provided as the basis of a new simulation. We simulated data in which the genetic contribution to baseline phenotype was determined solely by genotype at marker locus 2 on chromosome 21: if an individual had any copies of alleles 1–3 at this locus, the mean baseline phenotype was set to 5, otherwise it was set to 3. Similarly, the genetic contribution to slope phenotype was determined solely by genotype at marker locus 5 on chromosome 21: any copies of alleles 1–3 at this locus caused the slope value to have mean 0.002, otherwise mean 0.001. The final simulated glucose phenotype for individual *i *at time *t *was determined as

glucose = baseline + ε_1 _+ weight/150 + (slope + ε_2_)(age - 20) + ε_3_,

where at each *i *and *t*, ε_1_, ε_2 _and ε_3_, were sampled from normal distributions with mean 0 and standard deviations 0.4, 0.0001, and 0.0003, respectively. These data were analysed in the same way as the original data, and the results using replicate 1 only (1899 sib pairs) are shown in rows 1 and 2 of Figure 2. All four of the regression procedures succeeded in detecting and accurately locating both the locus controlling the baseline effect (which should be located at 13.84 cM) and the locus controlling slope effect (which should be located at 43.89 cM). It is interesting that the IBD regression method [4] gives higher significance than the other methods when detecting the baseline effect: further investigation of the properties of this method through theoretical calculations and simulation will be required to determine the cause of this elevated significance.

The success here could be due to the fact that the effects we simulated were relatively extreme, or to the fact that the generating model (equation (1)) corresponded to that assumed in the analysis. We therefore repeated the simulation using identical baseline and slope effects, but with the final glucose phenotype generating model altered to



(i.e., taking the same form as equation (2)) with ε_1_, ε_2_, and ε_3 _as described previously. The results are shown in rows 3 and 4 of Figure 2. Again we find that the loci controlling the baseline and slope effects are both accurately detected. It would therefore appear that our procedure works well even when the true biological model does not exactly correspond to that assumed in the derivation of the trait measures α and γ. The poor performance in the GAW13 simulated data is therefore likely to be due to the fact that in the GAW13 data, there were very many contributing genetic and environmental factors, resulting in a much smaller relative contribution for any given locus to the overall variation in fasting glucose. Unfortunately, for many complex traits the true situation is likely to be closer to the GAW13 simulation than to our simulation, indicating that often there may be very low power to detect effects of this magnitude, even with very large sample sizes.
